# Emotional Intelligence, Bullying, and Cyberbullying in Adolescents

**DOI:** 10.3390/ijerph16234837

**Published:** 2019-12-02

**Authors:** Inmaculada Méndez, Ana Belén Jorquera, Cecilia Ruiz-Esteban, Juan Pedro Martínez-Ramón, Aitana Fernández-Sogorb

**Affiliations:** 1 Department of Evolutionary Developmental and Educational Psychology, University of Murcia, 30100 Murcia, Spain; inmamendez@um.es (I.M.); anabelen.jorquera@um.es (A.B.J.); juanpedromartinezramon@um.es (J.P.M.-R.); 2 Department of Developmental Psychology and Didactics, University of Alicante, 03080 Alicante, Spain; aitana.fernandez@ua.es

**Keywords:** adolescence, bullying, cyberaggression, emotional intelligence, disruptive behavior

## Abstract

Bullying and cyberbullying are important global issues with negative consequences for physical and mental health in education. The objective of this study was to analyze to what extent some dimensions of emotional intelligence predict certain manifestations of bullying and cyberbullying in adolescents. The total number of subjects recruited in compulsory secondary education schools, was 309 (53.1% female). Their ages ranged from 12 to 16 (*M* = 14.17, *SD* = 1.4). The used instruments were the school violence questionnaire and the emotional coefficient inventory; the study design was cross-sectional. Results showed that the score increases on some scales (adaptability, stress management, and interpersonal) involved a greater risk of increasing the likelihood of social perception the different manifestations of school violence. However, in the general mood, the increase in this variable score implied lower perceiving in likelihood of violent behavior. It is important to take into account preventive actions aimed at improving school life and, above all, to alleviate difficulties in managing stress, adaptability, and interpersonal relationships.

## 1. Introduction

Bullying involves aggressive, intentional, and persistent behavior over time carried out by a group or individual against a victim who cannot easily defend themselves, so that there is a power imbalance. Three groups of key actors exist: aggressors, victims, and provocative victims [1]. Among manifestations of violence that can occur in educational contexts, we can highlight the following: physical violence (involving real contact to cause damage, directly or indirectly), verbal violence (implies the use of verbal language in an offensive way towards the victim, directly or covertly), social exclusion (acts of discrimination and rejection), disruption in the classroom (behaviors that make it difficult for teachers to teach classes), as well as violence through new technologies (cyberbullying, among others) [2,3]. Therefore, this classification includes certain manifestations of bullying and cyberbullying in adolescents, which is what we followed in our research [2]. The latest UNESCO report [4] warns that school violence and bullying are important global problems, and cyberbullying is also a growing problem. The report provides data from 144 countries, clarifying the most recent evidence on the magnitude, causes, and consequences of school violence and bullying. Globally, almost one in three students (32%) has been bullied at least once in the last month. With regard to Spain, the average prevalence of students who were bullied was 15.4%. The report issued by Save the Children in Spain [5] evidenced that 39.65% had suffered cyberbullying in their childhood, and 27.43% between once and twice. It should be noted that the data reported by the Foundation for the Help of Children and Adolescents at Risk (ANAR Foundation) [6] allege that the average number of violent acts suffered by victims of bullying has increased, with increasingly violent situations. In addition, 94% of the students who had been victims of bullying and 88.5% of victims of cyberbullying presented some psychological problem (for example, depression, anxiety, and fear).

The theoretical foundations of emotional intelligence (EI) have more recently been developed since the first contributions [7,8]. A systematic conceptual review was carried out. Accumulated evidence on EI has proven its existence from three different models: the performance-based ability model, the self-report ability model, and the self-report mixed model. According to the self-report mixed model, EI is not considered as a form of intelligence, but implies a broader concept that includes empathy, motivations, interpersonal/ intrapersonal skills, and personality [9]. Among self-report mixed models, we emphasize the Bar-on model [10,11], which is what we followed in our research. In this regard, an emotionally intelligent subject is one who is generally optimistic, realistic, flexible, able to solve problems successfully, and face stress without losing control [10]. We can also highlight the five following large dimensions: intrapersonal (self-awareness and self-expression), interpersonal (social awareness and interpersonal relationship), stress management (emotional management and regulation), adaptability (change management), and general mood (self-motivation) [11,12].

There are studies that show a link between EI and involvement in different forms of bullying. According to the systematic review carried out by García-Sancho et al. [13], an inverse relationship between EI and aggressive behavior was evidenced, regardless of sociocultural context, age, or type of aggression. Bullying is negatively correlated with general self-efficacy, emotional intelligence, emotional empathy, and their cognitive components [14]. In this vein, Lomas et al. [15] demonstrated that low management and emotional control were associated with high levels of victimization, and that understanding the emotions of others was negatively related with participation in bullying. Therefore, those adolescents with better emotional abilities have fewer negative emotions related to the expression of aggression or anger [16]. The existence of relationships between management and emotional control, and participation in internalization and outsourcing behaviors have been demonstrated by using nonproductive coping strategies to reduce stress instead of seeking a solution to the conflict [17], so aggressors had greater difficulties in the regulation and emotional management [18,19], and there was even an increased risk of family dysfunction [20]. Zych et al. [21] carried out a systematic review and found that cyberbullying perpetration was related to low empathy. It seems that perceived emotional intelligence acts as a moderating variable between cybervictimization and emotional impact by mitigating or increasing the different dimensions of emotional impact [22,23]. Along these lines, it has also been observed that EI deficits and its dimensions are positively associated with cybervictimization [24], especially to prevent them from being associated with other risk behaviors, such as drug consumption or involvement in antisocial behavior, as we have evidenced in previous studies conducted by the authors of this study [25,26].

Therefore, training or education in EI issues should be integrated into bullying and cyberbullying education. Regarding protective factors against bullying and cyberbullying [27], these are related to self-oriented personal competencies, the low frequency of technology use, good academic performance, and social skills oriented toward others, that is to say, the importance of positive interaction between partners. It is even essential to stimulate socially desirable and prosocial behavior, as well as to improve peer relationships [28]. Rodríguez-Hidalgo et al. [29] refer to the mitigation of ethical–cultural bullying. For this, it is necessary to prevent racism and xenophobia, and promote intercultural education. Likewise, it is essential to take into account family functioning for the positive development of students in order to minimize violent behavior [20], particularly due to the protective effect of parental control on cyberaggression, impulsivity, and high-risk Internet behaviors [30]. Smith et al. [31] pointed out the fundamental role of teachers in the prevention of bullying and cyberbullying, as well as the importance of improving security and climate in schools and other settings. Accordingly, Save the Children [5] alludes to the importance of training on the safe and responsible use of the Internet, with family–school collaborations and societal awareness, given the fact that interventions on violence in technology begin with information and understanding.

Meta-analyses carried out by Gaffney et al. indicated that antibullying programs reduce the perpetration of bullying by approximately 19–20%, and the victimization of bullying by about 15–16% [32]. Likewise, regarding cyberbullying, meta-analyses conducted by Gaffney et al. [33] indicate that intervention programs that have been carried out on cyberbullying are effective both in reducing the perpetration of cyberbullying (approximately 10–15%) and in cyberbullying victimization. Therefore training or education in EI issues should be integrated into bullying and cyberbullying education. It is necessary to implement programs to promote socioemotional development and prevent violence, as highlighted below. The programs promoted by Garaigordobil and Peña-Sarrionandia [34], and Garaigordobil and Martínez-Valderrey [35] showed an increase in empathic capacity, a decrease in bullying and cyberbullying, and an increase in cooperative conflict-resolution strategies. Thus, Sorrentino et al. [36] showed that the Tabby Improved Prevention Program, a multicomponent program developed by combining ecological-system theory and the threat-assessment approach, was effective in reducing cyberbullying and cybervictimization. The Asegúrate Program showed its effectiveness in reducing some forms of cyberbullying, clarifying the role of teachers in inclusion in ordinary curricula: the theory of normative social behavior, self-regulation skills, and beliefs held by adolescents, among others [37]. In addition, Ferrer-Cascales et al. [38] with the TEI Program, intervention based on peer tutoring, demonstrated a reduction of bullying and cyberbullying, as well as the improvement of school climate. The Prev@cib Bullying and Cyberbullying Program (2019), based on the ecological model, empowerment theory, and the model of personal and social responsibility, showed its effectiveness in reducing bullying and cyberbullying [39].

Therefore, the objective of this study was to analyze the extent to which some dimensions of emotional intelligence predict certain manifestations of bullying and cyberbullying in adolescents.

## 2. Materials and Methods

### 2.1. Participants

The total number of recruited participants in compulsory secondary education (CSE) schools was 309 (53.1% female) in different geographical areas of the region of Murcia (Spain). Ages ranged from 12 to 16 (*M* = 14.17, *SD* = 1.4), and gender distribution by age was homogeneous (*χ*^2^ = 3.64, *p* = 0.46).

First, an educational center was randomly selected from each of the northern, southern, eastern, and western areas of Murcia. Second, a class group was also randomly selected from each CSE course, recruiting 410 students of which 42 (10.4%) were dismissed for not providing the informed consent of parents or guardians, due to absence on the day of the questionnaire, or a substantial deficit in the use of the Spanish language, leaving 309 study participants.

The socioeconomic level of the areas and schools was average. The ethnic composition of the sample was: 91.72% Spanish, 4.89% Hispanic, 2.09% other European, 1.08% north Africa, and 0.22% Asian. Approximately, the 22.42% of the parents of the participating students had primary education, 55.74% had secondary education, 13.26% had university studies, and 8.58% did not report. The parental marital status: 67.04% married, 10.75% divorced or separated, 7.40% civil union, 3.05% single and the 11.76% did not report.

### 2.2. Instruments

The first scale was the school violence questionnaire (revised) [2]. It was composed of 31 Likert items with five response options: never, seldom, sometimes, often, and always. The questionnaire has eight factors: violence of teachers towards students (VTS), physical direct violence between students (VPD), physical indirect violence by students (VPI), verbal violence among students (VVS), verbal violence of students towards teachers (VVT), social exclusion (SE), disruptive behavior in the classroom (DB), and violence through new information and communication technologies (VICT). It has high reliability (α = 0.92), among study participants as well (α = 0.95). Example of items: “certain students send messages of offense, insult, or threat with their cell phones”; “students insult their classmates”.

Afterward, the emotional quotient-youth version (EQ-i: YV) by Bar-On and Parker was used, adapted to Spanish [12]. It comprised 60 items to assess the usage of a rate scale (from 1 = very seldom true or not true of me, to 4 = very often true of me or true of me). The five main dimensions are: intrapersonal (INTRA), interpersonal (INTER), stress management (SM), adaptability (ADAPT), and general mood (GM). Adequate reliability has been witnessed according to Cronbach’s alpha (α = 0.89) for this instrument [11] and in the application of this study (α = 0.86). Example of items: “I can be calm when I get angry”; I find it difficult to talk about my feelings to others”.

### 2.3. Procedure

The data of this work were collected as part of a broader project on intra and interpersonal variables that influence the teaching-learning. This is a cross-sectional descriptive study. The participants in this research were students selected from CSE schools in Murcia, Spain. For data collection, firstly, an initial telephone contact was established with the directors of the schools, followed by a meeting was carried out with the principals and school psychologists of the participating schools in order to present the objectives of the research, describe the assessment instruments (instructions of each of the instruments) and request permission. To encourage their cooperation, they were also told that a report with the results of the study would be delivered to each center with results-based guidance. After obtaining permission, students were approached at their own classrooms in school. The questionnaires were filled out voluntarily during a classroom session. The instructions were read aloud, emphasizing the importance of answering all questions. The researchers were present during the administration of the tests to resolve doubts and ensure an unbiased process. Participation was anonymous.

### 2.4. Data Analysis

First, the school violence questionnaire (revised) (CUVE-R) variables were dichotomized into high scores (≥percentile 75) and low scores (≤percentile 25). The logistic regression method was used following the stepwise regression procedure based on the Wald statistic to analyze the predictive capacity of EI factors on high scores in school violence. This prediction capability was estimated by the odds ratio (OR) statistic that is interpreted as follows: OR > 1 indicates prediction in a positive direction, OR < 1 indicates prediction in the negative, while a value of 1 indicates that there is no prediction [40]. OR and a 95% confidence interval were calculated in each case. The Hosmer and Lemeshow (HL) test was used to verify the fit quality of the model [41]. All analyses were performed with SPSS 24.0 (IBM, Armonk, NY, USA).

### 2.5. Ethics Approval

The study protocols were approved by the Ethics Committee for Clinical Investigations of the University of Murcia. Moreover, the study was performed in accordance with approved guidelines and the Declaration of Helsinki, with written informed consent from all participants.

## 3. Results

Table 1 shows the variables included in each of the eight models for the logistic-regression procedure. First, simple logistic-regression analysis enabled us to detect the individual effect of each variable in emotional intelligence. (1) The violence of teachers towards students model correctly classified 70.7% of the cases, and the adjustment value was *R*^2^ = 0.20; in this, for each point of increase in adaptability (OR = 1.10) and in stress management (OR = 1.15), students were at greater risk of perceive violence of teachers towards students, but not in general mood, since the probability of violence of teachers towards students (OR = 0.92) decreased. (2) The physical indirect violence by students model correctly classified 65.3% of the cases, and the adjustment value was *R*^2^ = 0.17; moreover, for each point of increase in stress management (OR = 1.17), students presented a higher risk of perceiving physical indirect violence by students, but not in general mood since such violence decreased (OR = 0.94). (3) The physical direct violence between students model correctly classified 64.5% of the cases, and the model fit was *R*^2^ = 0.09; in this one, for each point of increase in stress management (OR = 1.11), students were more perceiving physical direct violence between students. (4) The verbal violence among students model correctly classified 70.9% of the cases, and the adjustment value was *R*^2^ = 0.27; in this, for each point of increase in adaptability (OR = 1.11) and in stress management (OR = 1.18), students were more perceiving verbal violence among students, but not in general mood since it decreased (OR = 0.90). (5) The verbal violence of students towards teachers model correctly classified 71.8% of the cases, and the adjustment was *R*^2^ = 0.18; in this model, for each point of increase in stress management (OR = 1.10), students were more at risk of perception of verbal violence of students towards teachers, but not in general mood (OR = 0.96). (6) The social exclusion model correctly classified 70.9% of the cases, and the adjustment value was *R*^2^ = 0.16, in which students, for each point of increase in stress management (OR = 1.17), presented a higher risk of perceiving social exclusion. (7) The disruptive behavior in the classroom model correctly classified 68.2% of the cases, adjustment value was *R*^2^ = 0.15, and for each point of increase in interpersonal (OR = 1.11) and stress management (OR = 1.12), students presented a higher risk of perceiving disruptive behavior in the classroom, but not in general mood (OR = 0.94). (8) The violence through new information and communication technologies model correctly classified 67.6% of the cases, and the adjustment value was *R*^2^ = 0.22; in this one, for each point of increase in stress management (OR = 1.18), students were more at risk of perceiving violence through new information and communication, but not in general mood (OR = 0.94).

## 4. Discussion

In order to meet the EI dimensions that underlie involvement in bullying and cyberbullying, it was necessary to conduct analysis as a whole. In this regard, after the study was carried out, we could deduce that the increase in the general mood score implied lower perceive as violence of teachers towards students, physical indirect violence by students, verbal violence among students, verbal violence of students towards teachers, disruptive behavior in the classroom, and violence through new information and communication technologies. This means that those people who are usually optimistic, positive, and generally pleasant to be with are less perceive in the mentioned forms of school violence. Therefore, those adolescents with better emotional abilities have fewer negative emotions related to the expression of aggressiveness or anger [13,14,16,20] due to the moderating role of EI [23].

Regarding stress management, it is noteworthy that an increase in this variable implies an increase in all variables of measured school violence (violence of teachers towards students, physical indirect violence by students, physical direct violence between students, verbal violence among students, verbal violence of students towards teachers, social exclusion, disruptive behavior in the classroom, and violence through new information and communication technologies). Hence, students with difficult stress management scores are usually people with some inability to relax and irritability. They usually use nonproductive coping strategies to reduce stress instead of seeking solutions to conflict [17], presenting greater difficulties in emotional regulation and management [18,19,20,24].

It is worth mentioning that, for each interpersonal increase, probability of disruptive behavior perceived in the classroom increased. The more difficulty the students had interacting with others, since they may have difficulties in identifying emotions in others, the greater involvement in disruptive behaviors [13,15,16,19,20].

However, we must emphasize that, for each increase in the adaptability score, the risk of students perceiving high scores in violence of teachers towards students and verbal violence among students increased. They are inflexible people, usually lack the necessary skills to manage changes, and it is difficult for them to solve the problems of each day without engaging in aggressive behaviors [17,20].

As far as limitations to the study are concerned, it should be noted that results may be difficult to generalize with students of other educational levels; the data of this research are based exclusively on measures of self-report [9], a fact that can introduce biases derived from the social desirability of the participants. In another vein, this is a cross-sectional study, so it would be interesting to carry out longitudinal studies as well as increase the sample. Additional research is needed to examine other factors that may exacerbate these situations in adolescence.

## 5. Conclusions

Results showed that the score increases on some scales (adaptability, stress management, and interpersonal) involved a greater risk of increasing the likelihood of perceive the different manifestations of school violence. Not so in the general mood, the increase in this variable score implied lower perceived in in likelihood of violent behavior. Therefore, the results of the study allow taking into account preventive actions aimed at improving school life, alleviating difficulties in stress management, adaptability, and interpersonal relationships [14,15,17,18,19,20,24], and, above all, to prevent them from being associated with other risky behaviors, such as drug consumption or involvement in antisocial behavior [30,31].

Therefore, training or education in EI issues should be integrated into bullying and cyberbullying education. Among preventive actions, emotional regulation can be considered a valuable resource [24]; self-oriented personal competencies [27], stimulating socially desirable and prosocial behavior [28,35,39]; peer tutoring [38]; mitigation of ethno–cultural bullying [29]; it is essential to take into account family functioning for the positive [20,30]; the fundamental role of teachers in the prevention of bullying and cyberbullying; as well as the importance of improving security and climate in schools and other settings [31,37]. According with Save the Children [5] it is everyone’s responsibility to eradicate violence.

Finally, we conclude that bullying and cyberbullying can have a significant impact on mental health, quality of life, and risky behaviors [4]. Thus, bullying and cyberbullying have devastating effects on victims. Academic consequences may have an impact on future prospects and employability [4]. Such harassment can even transcend the university stage [42,43,44]. These are social and public health problems [35]. For this reason, school violence in all its manifestations is a violation of the rights of children and adolescents in education, health, and well-being. Under the circumstances, it is imperative to eradicate school violence and bullying to achieve inclusive and equitable quality. Focusing on school climate, classroom management and discipline, emotional skills, and the relationship between teachers and students, and among students can help reduce bullying and violence, and their negative impact on learning outcomes. Because of all this, the Safe to Learn Campaign aims to end all violence in schools by 2024 by raising awareness and catalyzing actions to eliminate school violence and bullying. Addressing school violence and bullying is crucial in order to achieve sustainable development goals (SDGs) [4].

## Figures and Tables

**Table 1:** Binary logistic regression for the probability of high scores on school–violence factors depending on emotional intelligence.

Variable		χ^2^	*R* ^2^	B	S.D.	Wald	*p*	OR	C.I. 95%	H.L.
VTS	C.C. 70.7%	25.21	0.20							χ^2^ = 12.90, *p* = 0.12
	ADAPT			0.10	0.04	4.96	0.026	1.10	1.01–1.20	
	SM			0.14	0.04	12.90	0.000	1.15	1.07–1.25	
	GM			−0.08	0.03	8.37	0.004	0.92	0.87–0.97	
	Constant			−2.44	1.50	2.83	0.093	0.09		
VPI	C.C. 65.3%	28.39	0.17							χ^2^ = 7.40, *p* = 0.50
	SM			0.15	0.04	18.81	0.000	1.17	1.09–1.25	
	GM			−0.06	0.02	8.25	0.004	0.94	0.91–0.98	
	Constant			−1.31	1.16	1.29	0.256	0.27		
VPD	C.C. 64.5%	11.88	0.09							χ^2^ = 4.17, *p* = 0.84
	SM			0.11	0.03	10.52	0.001	1.11	1.04–1.19	
	Constant			−2.56	0.88	8.43	0.004	0.08		
VVS	C.C. 70.9%	40.07	0.27							χ^2^ = 3.86, *p* = 0.87
	ADAPT			0.10	0.04	7.50	0.006	1.11	1.03–1.19	
	SM			0.17	0.04	19.64	0.000	1.18	1.10–1.27	
	GM			−0.10	0.03	15.52	0.000	0.90	0.86–0.95	
	Constant			−2.38	1.32	3.27	0.070	0.09		
VVT	C.C. 71.8%	18.39	0.08							χ^2^ = 13.23, *p* = 0.10
	SM			0.10	0.03	13.02	0.000	1.10	1.05–1.16	
	GM			−0.04	0.02	5.34	0.021	0.96	0.93–0.99	
	Constant			−1.74	0.99	3.09	0.079	0.18		
SE	C.C. 70.9%	20.26	0.16							χ^2^ = 10.29, *p* = 0.25
	SM			0.16	0.04	16.11	0.000	1.17	1.08–1.26	
	Constant			−3.57	1.02	12.24	0.000	0.03		
DB	C.C. 68.2%	20.60	0.15							χ^2^ = 9.01, *p* = 0.34
	INTER			0.10	0.03	9.99	0.002	1.11	1.04–1.18	
	SM			0.11	0.04	8.95	0.003	1.12	1.04–1.20	
	GM			−0.06	0.03	5.34	0.021	0.94	0.90–0.99	
	Constant			−3.77	1.78	4.48	0.034	0.02		
VICT	C.C. 67.6%	31.79	0.22							χ^2^ = 9.82, *p* = 0.28
	SM			0.16	0.04	19.88	0.000	1.18	1.10–1.27	
	GM			−0.06	0.02	8.08	0.004	0.94	0.90–0.98	
	Constant			−1.39	1.24	1.27	0.260	0.25		

ADAPT: adaptability. B: regression coefficient. C.C.: classified correctly. C.I.: 95% confidence interval. DB: disruptive behavior in the classroom. INTER: interpersonal. INTRA: intrapersonal. GM: general mood. OR: odds ratio. *p*: probability. *R*^2^: Nagelkerke’s R squared. HL: Hosmer and Lemeshow. SE: social exclusion. S.D.: standard deviation. SM: stress management. VICT: violence through new information and communication technologies. VPD: physical direct violence between students. VPI: physical indirect violence by students. VTS: violence of teacher towards students. VVS: verbal violence among students. VVT: verbal violence of students towards teachers. Wald = Wald test. χ^2^: Chi squared test.
